# How does antenatal lifestyle affect the risk for gestational diabetes mellitus? A secondary cohort analysis from the GeliS trial

**DOI:** 10.1038/s41430-021-00910-9

**Published:** 2021-04-23

**Authors:** Julia Günther, Julia Hoffmann, Lynne Stecher, Monika Spies, Kristina Geyer, Roxana Raab, Dorothy Meyer, Kathrin Rauh, Hans Hauner

**Affiliations:** 1 Else Kröner-Fresenius-Centre for Nutritional Medicine, Klinikum rechts der Isar, Technical University of Munich, Munich, Germany; 2 Competence Centre for Nutrition (KErn), Freising, Germany

**Keywords:** Risk factors, Metabolic disorders, Gestational diabetes

## Abstract

**Objectives:**

We aimed to investigate the predictive potential of early pregnancy factors such as lifestyle, gestational weight gain (GWG) and mental well-being on gestational diabetes mellitus (GDM) beyond established risk factors.

**Methods:**

GDM risk was investigated in the cohort of the German ‘Gesund leben in der Schwangerschaft’/healthy living in pregnancy study. Women were recruited up to the 12^th^ week of gestation. GDM was diagnosed with a 75 g oral glucose tolerance test between the 24^th^ and 28^th^ weeks of gestation. Pre-pregnancy age and weight, mental health and lifestyle were assessed via questionnaires. Maternal weight was measured throughout pregnancy. Early excessive GWG was defined based on the guidelines of the Institute of Medicine. The association between several factors and the odds of developing GDM was assessed using multiple logistic regression analyses.

**Results:**

Of 1694 included women, 10.8% developed GDM. The odds increased with pre-pregnancy BMI and age (women with obesity: 4.91, CI 3.35–7.19, *p* < 0.001; women aged 36–43 years: 2.84, CI 1.45–5.56, *p* = 0.002). Early excessive GWG, mental health and general lifestyle ratings were no significant risk factors. A 31% reduction in the odds of GDM was observed when <30% of energy was consumed from fat (OR 0.69, CI 0.49–0.96, *p* = 0.026). Vigorous physical activity tended to lower the odds without evidence of statistical significance (OR 0.59 per 10 MET-h/week, *p* = 0.076).

**Conclusions:**

Maternal age and BMI stand out as the most important drivers of GDM. Early pregnancy factors like dietary fat content seem to be associated with GDM risk. Further evaluation is warranted before providing reliable recommendations.

## Introduction

The prevalence of gestational diabetes mellitus (GDM) has considerably increased over the last few years [1, 2]. This trend is alarming as GDM increases the risk of maternal and offspring complications, including caesarean section and macrosomia [3]. Furthermore, a dramatically increased long-term risk of type 2 diabetes in mothers [2] and early-onset of obesity in the offspring [4] are discussed.

The development of GDM is putatively influenced by several determinants, including maternal age and pre-pregnancy body weight status [5]. Beyond these, various modifiable influencing factors have been suggested. In particular, early excessive gestational weight gain (GWG) was found to be positively associated with development of GDM [6].

There is evidence that lifestyle factors, including dietary and physical activity behaviour, can modify GDM risk [7, 8]. However, research remains inconclusive. Moreover, studies are often based on limited data sets focusing on the potential impact of certain lifestyle variables alone [7, 8]. As many potential risk factors are discussed [9–11], well-defined cohorts with diverse data sets are urgently required for further elucidating the complex interaction of these risk factors and ultimately for the development of effective prevention strategies.

The large-scaled, cluster-randomised, controlled GeliS study (‘Gesund leben in der Schwangerschaft’/healthy living in pregnancy) sought to prevent excessive GWG and associated complications including GDM. Data on the effects on GWG, GDM incidence, maternal and infant health, and lifestyle have recently been published [12–16]. The GeliS cohort offers comprehensive data on lifestyle and maternal and offspring health and thus provides the opportunity to analyse the predictive potential of diverse factors on GDM development. This secondary analysis aimed to investigate associations between the incidence of GDM and maternal socio-demographics, early pregnancy lifestyle, GWG and mental health before GDM screening.

## Subjects and methods

### Study design

The cluster-randomised, controlled GeliS trial was conducted within the German routine health care system in five regions of Bavaria, Germany, as described in the published study protocol [17].

The study was conducted in accordance with the Declaration of Helsinki and local regulatory requirements and laws. The study protocol was approved by the ethics committee of the Technical University of Munich and is registered in the ClinicalTrials.gov Registration System (NCT01958307).

### Participants

Pregnant women aged between 18 and 43 years with a pre-pregnancy BMI between 18.5 and 40.0 kg/m^2^ were recruited in gynaecological and midwifery practices until the 12^th^ week of gestation between 2013 and 2015. Women provided written informed consent for participation. They were excluded from participation in case of a multiple or complicated pregnancy or severe illness [17].

### Procedures

Participants in the control group obtained routine prenatal care and leaflets with general recommendations on a healthy antenatal lifestyle. Women in the intervention group additionally received structured lifestyle counselling.

The counselling consisted of three individual antenatal and one postpartum sessions, including standardised information on adequate GWG, a balanced diet and regular physical activity during pregnancy according to national and international recommendations [18, 19]. After training by the study team, midwives, gynaecologists and medical assistants conducted the sessions during routine appointments. Details on the lifestyle intervention programme have been reported previously [17].

### Outcomes and data collection

The primary outcome of the GeliS trial was the proportion of women showing excessive GWG as defined by the Institute of Medicine (IOM) [20]. Despite some alterations in antenatal dietary and physical activity behaviour [13, 14], no considerable between-group differences in maternal and neonatal weight and health outcomes including GDM incidence were found [12]. Thus, data from the groups were pooled to form one cohort for the present analysis.

GDM status was assessed via a standardised 2-h 75 g oral glucose tolerance test (oGTT) between the 24^th^ and 28^th^ weeks of gestation. Tests were performed in gynaecological practices according to national and international guidelines [21, 22]. GDM was diagnosed if one or more of the following thresholds was met or exceeded: Fasting plasma glucose: 92 mg/dL (5.1 mmol/L), 1 h: 180 mg/dL (10.0 mmol/L) and 2 h: 153 mg/dL (8.5 mmol/L).

At study entry, women reported demographic data and pre-pregnancy weight in a screening questionnaire. BMI categorisation was based on pre-pregnancy weight as reported in this questionnaire. During the course of pregnancy, weight was continuously measured in gynaecological or midwife practices and documented in maternity records. Early GWG was defined as the difference between maternal weight measured in the second trimester (between the 16^th^ and the 20^th^ weeks of gestation) and the weight measured at the first prenatal visit prior the 12th week of gestation. Early excessive GWG was defined according to the recommendations of the IOM [20]. For the first trimester, it is advised not to exceed 2 kg of weight gain. For the second trimester, optimal GWG is defined in relation to weekly GWG depending on pre-pregnancy BMI category. Herein, early excessive GWG was calculated considering the timing of weight measurement per participant, according to Hedderson et al. [23].

Data on pre-pregnancy and early pregnancy lifestyle, such as smoking status, dietary behaviour, physical activity and mental health, were collected via questionnaires until the 12^th^ week of gestation. Dietary behaviour was assessed with a validated Food Frequency Questionnaire (FFQ) [24]. Details on the assessment and evaluation have been described in detail elsewhere [13]. Dietary quality was rated with a Healthy Eating Index (HEI) that was specifically developed for the utilised FFQ [13, 25]. The HEI had a maximum score of 100. Median HEI was used to group participants into meeting a ‘low HEI’ or a ‘high HEI’. Physical activity was evaluated via the validated Pregnancy Physical Activity Questionnaire [26]. Details on the assessment and evaluation have been described in detail previously [14]. Median ‘physical activity of light intensity and above’ was used to group participants into ‘low physical activity’ or ‘high physical activity’ categories.

Mental well-being and depression were assessed using the German version of the Patient Health Questionnaire-4 (PHQ-4) and the World Health Organization Well-Being Index (WHO-5) [27]. A PHQ-4 score of at least 3 on the 12-point scale was used as a cut-off to group women as probable and improbable cases of depression or anxiety [28]. A WHO-5 score lower than 50% was used to group participants as having low antenatal well-being [28].

Participants were grouped into having a ‘lower educational level’ if they had completed general secondary education and into the ‘higher educational level’ category if they had completed intermediate secondary school or high school. Participants were grouped into ‘never smoking’ or ‘ever smoking’.

### Statistical analysis

Participants were included in the analyses if they underwent the standardised oGTT and if socioeconomic data and valid lifestyle information were available. Women who dropped out during pregnancy were excluded. Intervention and control groups were pooled to form one cohort. Baseline characteristics are presented as mean ± standard deviation or proportions if appropriate. Descriptive data were stratified by GDM status and statistical differences between those with and without GDM were assessed using the χ^2^ test for categorial variables and the Kruskal–Wallis test for continuous variables.

Multivariate logistic regression models were fitted to examine associations between potential predictor variables and GDM incidence. Four regression models were applied with the addition of covariates in each model. Model 1 included pre-pregnancy BMI, age and group allocation as covariates. Model 2 additionally considered early excessive GWG and nulliparity. Model 3 was further adjusted for smoking status, low dietary quality, and low physical activity. Model 4 additionally considered educational level and antenatal depression. The fully adjusted model (Model 4) was exploratorily extended by an interaction term between pre-pregnancy BMI and early excessive GWG.

Potential associations between more specific dietary and physical activity variables and GDM incidence were exploratorily tested using further logistic regression models, adjusted for pre-pregnancy BMI, age, parity and group allocation.

*P* values below 0.05 were considered as statistically significant. All analyses were performed with SPSS software (IBM SPSS Statistics for Windows, version 24.0, IBM Corp, Armonk, NY, USA). Power calculation was based on the primary outcome of the original intervention study [17]. Due to the exploratory character of this secondary analysis, no adjustment for multiple testing was performed.

## Results

### Flowchart and baseline characteristics of participants

**Fig. 1:**
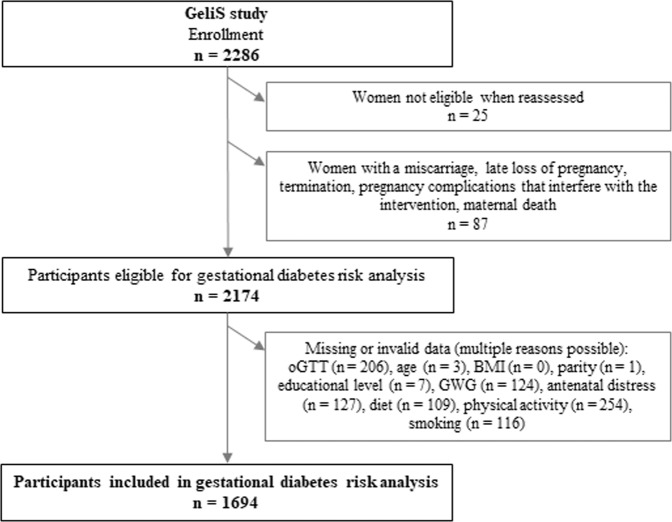
Flowchart of women enrolled in the GeliS trial and considered in gestational diabetes risk analysis. BMI body mass index, GeliS ‘Gesund leben in der Schwangerschaft’/healthy living in pregnancy, GWG gestational weight gain, oGTT oral glucose tolerance test.

The flow of participants in the GeliS trial is presented in Fig. 1. Initially, 2286 women were recruited for study participation. Of the 2174 women potentially eligible for the GDM analysis, 1694 women were included in the predictor analysis.

An overview of maternal characteristics, categorised by GDM diagnosis, is given in Table 1. Total GDM incidence was 10.8%. In the group of women with GDM, 23.0% were characterised as having pre-pregnancy overweight and 31.7% as having pre-pregnancy obesity. Among women with a negative test result, the rates of pre-pregnancy overweight and obesity were 22.7% and 9.8% (*p* < 0.001), respectively. Mean self-reported pre-pregnancy weight and age were higher in the group with GDM compared to the group without GDM diagnosis (75.4 ± 16.1 kg vs. 67.4 ± 12.8 kg, and 31.4 ± 4.5 years vs. 30.2 ± 4.4 years, *p* < 0.001 for both comparisons). The remaining parameters were comparable between the two groups.

**Table 1:** Characteristics of study participants with positive and negative GDM diagnosis.

	No GDM (*n* = 1511, 89.2%)	GDM (*n* = 183, 10.8%)	Total (*n* = 1694)	*p* value^a^
*Group allocation (n (%))*				0.880
Control group	719/1511 (47.6%)	86/183 (47.0%)	805/1694 (47.5%)	
Intervention group	792/1511 (52.4%)	97/183 (53.0%)	889/1694 (52.5%)	
*Pre-pregnancy age (years)*^b^	30.2 ± 4.4	31.4 ± 4.5	30.4 ± 4.4	<0.001
*Pre-pregnancy weight (kg)*	67.4 ± 12.8	75.4 ± 16.1	68.3 ± 13.4	<0.001
*Pre-pregnancy BMI (kg/m*^*2*^*)*	24.1 ± 4.2	27.1 ± 5.6	24.4 ± 4.5	<0.001
*Pre-pregnancy BMI category (n (%))*				<0.001
BMI 18.5–24.9 kg/m^2^	1020/1511 (67.5%)	83/183 (45.4%)	1103/1694 (65.1%)	
BMI 25.0–29.9 kg/m^2^	343/1511 (22.7%)	42/183 (23.0%)	385/1694 (22.7%)	
BMI 30.0–40.0 kg/m^2^	148/1511 (9.8%)	58/183 (31.7%)	206/1694 (12.2%)	
*Educational level (n (%))*				0.563
General school^c^	204/1511 (13.5%)	30/183 (16.4%)	234/1694 (13.8%)	
Vocational secondary school	655/1511 (43.3%)	77/183 (42.1%)	732/1694 (43.2%)	
Academic high school	652/1511 (43.2%)	76/183 (41.5%)	728/1694 (43.0%)	
*Country of birth (n (%))*				0.854
Germany	1354/1509 (89.7%)	165/183 (90.2%)	1519/1692 (89.8%)	
Others	155/1509 (10.3%)	18/183 (9.8%)	173/1692 (10.2%)	
*Nulliparous (n (%))*	886/1511 (58.6%)	107/183 (58.5%)	993/1694 (58.6%)	0.966
*Living with a partner (n (%))*	1454/1506 (96.5%)	176/183 (96.2%)	1630/1689 (96.5%)	0.796
*Full-time employed (n (%))*	800/1502 (53.3%)	101/179 (56.4%)	901/1681 (53.6%)	0.423
*Current or former smoker (n (%))*	715/1511 (47.3%)	97/183 (53.0%)	812/1694 (47.9%)	0.146
*Low HEI*^d^ *(n (%))*	677/1511 (44.8%)	87/183 (47.5%)	764/1694 (45.1%)	0.482
*Low PA*^e^ *(n (%))*	743/1496 (49.7%)	93/181 (51.4%)	836/1677 (49.9%)	0.663
*Excessive GWG until oGTT (n (%))*	920/1511 (60.9%)	107/183 (58.5%)	1027/1694 (60.6%)	0.527
*Antenatal distress*^f^ *(n (%))*	641/1511 (42.4%)	76/183 (41.5%)	717/1694 (42.3%)	0.818
*Low well-being*^g^ *(n (%))*	546/1496 (36.5%)	68/181 (37.6%)	614/1677 (36.6%)	0.777

*BMI* body mass index, *GDM* gestational diabetes mellitus, *GWG* gestational weight gain, *HEI* Healthy Eating Index, *oGTT* oral glucose tolerance test, *PA* physical activity, *PHQ-4* Patient Health Questionnaire-4, *SD* standard deviation, *WHO-5* World Health Organization Well-Being Index 5.

^a^*p* value for differences between women with and without GDM, tested with χ^2^ test for categorial variables and Kruskal–Wallis test for continuous variables.

^b^Mean ± SD (all such values).

^c^School is completed through year 9.

^d^HEI below the 50th percentile.

^e^Total physical activity below the 50th percentile.

^f^PHQ-4 score of ≥3 points.

^g^WHO-5 score <50%.

### Analysis of early GDM predictors

Table 2 shows the results of the GDM risk factor logistic regression analysis. Maternal pre-pregnancy BMI and age category were positively associated with the odds of developing GDM (Model 1: OR for women with overweight: 1.51, 95% CI 1.02–2.24, *p* = 0.039; OR for women with obesity: 4.91, 95% CI 3.35–7.19, *p* < 0.001; OR for women aged 26–35 years: 2.09, 95% CI 1.17–3.73, *p* = 0.013; OR for women aged 36–43 years: 2.84, 95% CI 1.45–5.56, *p* = 0.002). The addition of the variable early excessive GWG did not change the relationships between either pre-pregnancy BMI or age category and the odds of developing GDM (Model 2: *p* = 0.749). There were no significant associations between early pregnancy lifestyle factors including low HEI (*p* = 0.530), low physical activity (*p* = 0.916) and smoking (*p* = 0.417) and the odds of developing GDM (Model 3). In the fully adjusted model, there was no statistical evidence that early pregnancy anxiety/distress (*p* = 0.986), or low maternal education (*p* = 0.739) had an impact on GDM incidence. The association between age and pre-pregnancy BMI and GDM incidence remained statistically significant in all models. Substituting antenatal distress/anxiety by a low well-being status neither provided evidence for a predictive potential nor changed the described associations (data not shown). In the fully adjusted model (Model 4), there was no evidence that an interaction between maternal pre-pregnancy BMI and early excessive GWG impacted the odds of having GDM (data not shown). Including BMI, age and other continuous covariates as continuous linear variables in the model did not notably change the results (Supplementary Table S1).

**Table 2:** Associations between demographic and lifestyle factors and the odds of developing GDM.

Covariate	Model 1	Model 2	Model 3	Model 4
*Group allocation*	1.03 (0.75–1.41)	1.02 (0.74–1.40)	1.02 (0.74–1.41)	1.02 (0.74–1.41)
*BMI category*^a^				
BMI 25.0–29.9 kg/m^2^	1.51 (1.02–2.24)^+^	1.53 (1.03–2.26)^+^	1.51 (1.02–2.24)^+^	1.50 (1.01–2.24)^+^
BMI 30.0–40.0 kg/m^2^	4.91 (3.35–7.19)^+++^	4.88 (3.33–7.16)^+++^	4.66 (3.16–6.87)^+++^	4.63 (3.13–6.84)^+++^
*Pre-pregnancy age category*^b^				
26–35 years	2.09 (1.17–3.73)^+^	2.12 (1.18–3.80)^+^	2.20 (1.22–3.97)^++^	2.22 (1.22–4.03)^++^
36–43 years	2.84 (1.45–5.56)^++^	2.95 (1.49–5.82)^++^	2.98 (1.50–5.94)^++^	3.00 (1.50–5.99)^++^
*Nulliparity*		1.10 (0.79–1.52)	1.08 (0.76–1.53)	1.08 (0.77–1.53)
*Early excessive GWG*^c^		0.95 (0.69–1.31)	0.91 (0.66–1.27)	0.91 (0.66–1.27)
*Low HEI*^d^			1.11 (0.80–1.53)	1.10 (0.80–1.53)
*Low PA*^e^			1.02 (0.73–1.42)	1.02 (0.73–1.43)
*Smoking*^f^			1.14 (0.83–1.58)	1.14 (0.82–1.58)
*Low education*^g^				1.08 (0.69–1.69)
*Antenatal anxiety/distress*^h^				1.00 (0.72–1.38)

*BMI* body mass index, *GDM* gestational diabetes mellitus, *GWG* gestational weight gain, *HEI* Healthy Eating Index, *PA* physical activity, *PHQ-4* Patient Health Questionnaire-4.

^+^*p* < 0.05.

^++^*p* < 0.01.

^+++^*p* < 0.001.

^a^BMI 18.5–24.9 kg/m^2^ was used as reference.

^b^Age 18–25 years was used as reference.

^c^Defined according to the Institute of Medicine (IOM).

^d^HEI below the 50^th^ percentile.

^e^Total physical activity below the 50^th^ percentile.

^f^Current or former smokers.

^g^General secondary education or lower.

^h^PHQ-4 score of ≥3 points.

### Associations between specific dietary and physical activity variables and GDM risk

Table 3 summarises associations between specific dietary and physical activity variables and the odds of developing GDM. While energy intake of women was not associated with the odds of GDM (*p* = 0.736), there was significant evidence of a link between specific macronutrient patterns and GDM development. A diet with less than 30% of energy (E%) originating from fat was associated with a 31% reduction in the odds of GDM (OR 0.69, CI 0.49–0.96, *p* = 0.026). The odds of developing GDM increased by 3% per E% consumed from fat (OR 1.03, CI 1.00–1.05, *p* = 0.040). The same non-significant trend was observed when only saturated fat was considered (OR 1.05, CI 1.00–1.10, *p* = 0.055). Correspondingly, a low proportion of energy from carbohydrates (<50 E%) was by trend, but not significantly, associated with an increase in the odds of GDM (OR 1.44, CI 0.99–2.09, *p* = 0.058).

**Table 3:** Associations between specific dietary and physical activity variables and the odds of developing GDM.

	*n*	Gestational diabetes mellitus OR (CI)^a^	*p* value^a^
*Energy and macronutrient intake*			
Energy intake (per 100 kcal/day)	1686	1.00 (0.97–1.02)	0.736
E% fat (per E%/day)	1686	1.03 (1.00–1.05)	0.040
E% saturated fat (per E%/day)	1686	1.05 (1.00–1.10)	0.055
Low fat diet (<30 E%/day)	1686	0.69 (0.49–0.96)	0.026
E% protein (per E%/day)	1686	1.01 (0.96–1.06)	0.739
Carbohydrates (<50 E%/day)	1686	1.44 (0.99–2.09)	0.058
Sugar intake (per g/day)	1686	1.00 (1.00–1.01)	0.431
Fibre (per g/day)	1686	0.99 (0.98–1.01)	0.264
*Food intake*			
Soft drinks (200 ml/day)	1855	0.97 (0.92–1.03)	0.295
Sweets and snacks (50 g/day)	1856	1.02 (0.89–1.15)	0.813
Dairy products (200 g/day)	1855	1.08 (1.01–1.17)	0.035
Meat and meat products (150 g/day)	1855	0.76 (0.51–1.16)	0.205
Fast food (250 g/day)	1855	0.57 (0.18–1.78)	0.330
*Physical activity*^b^			
Sedentary behaviour	1838	1.11 (0.97–1.27)	0.118
Moderate intensity physical activity	1796	1.01 (0.98–1.04)	0.577
Vigorous-intensity physical activity	1844	0.59 (0.33–1.06)	0.076
Sports	1824	0.87 (0.73–1.04)	0.133

*BMI* body mass index, *CI* confidence interval, *E%* energy percent, *GDM* gestational diabetes mellitus, *MET* metabolic equivalent of task, *OR* odds ratio.

^a^Adjusted for maternal pre-pregnancy BMI, age, parity and group assignment.

^b^Effect sizes are calculated per 10 MET-h/week.

Among specific food groups, the group of milk and dairy was the only one significantly related to the odds of developing GDM (8% increase per 200 g portion per day, CI 1.01–1.17, *p* = 0.035). Consumption of soft drinks, sweets, meat and fast food was not significantly associated with GDM (Table 3). An overview of mean food intake and physical activity of women grouped according to GDM diagnosis is given in Supplementary Table S2.

Engaging in vigorous-intensity physical activity was, by trend, inversely related to the odds of GDM (OR 0.59 per 10 MET-h/week, CI 0.33–1.06, *p* = 0.076). None of the other analysed physical activity variables showed a significant association with the odds of GDM.

An overview of mean food intake and physical activity of women grouped according to GDM diagnosis is given in Supplementary Table S2.

## Discussion

In this secondary cohort analysis from the GeliS study, maternal age and pre-pregnancy BMI were identified as the main pre-pregnancy predictors of GDM. While overall socio-demographic and lifestyle factors were not associated with GDM risk in the fully adjusted prediction model, some specific dietary variables, including macronutrient composition, were significantly associated with GDM incidence.

Increased maternal age and pre-pregnancy overweight or obesity are established risk factors of impaired glucose tolerance during pregnancy [5]. However, evidence on the role of excessive GWG is inconclusive [20]. In contrast to findings from a recent meta-analysis [6], we could not confirm that early excessive GWG significantly contributed to the risk of developing GDM. It is well described that GWG is highly influenced by maternal lifestyle during the course of pregnancy [13, 14, 29]. However, little is known about the complex interaction between GWG and modifiable lifestyle factors, such as dietary and physical activity patterns, on GDM risk, and reported findings are inconclusive [7, 20]. We did not observe that lifestyle variables, including ratings of low overall dietary quality, low total physical activity, maternal smoking and low mental well-being, notably changed relationships between maternal age and pre-pregnancy BMI on GDM incidence. However, in additional analyses, vigorous physical activity in early pregnancy was associated with decreased GDM risk by non-significant trend. These findings are similar to observations from the Nurses’ Health Study II, which reported associations between lowered GDM risk and pre-pregnancy physical activity [9]. We were unable to confirm observations from a meta-analysis [8], in which overall pre- and early pregnancy physical activity resulted in a general reduction of GDM risk. Notably, and in contrast to our analyses, studies included in this meta-analysis did not consistently correct for maternal age and pre-pregnancy BMI. Further adjustment for maternal BMI by the authors of the meta-analysis weakened the reported overall association between physical activity and GDM risk [8].

Although overall low dietary quality was not significantly associated with GDM risk in our prediction model, additional analyses elucidated the contribution of single dietary components. In particular, dietary fat content was associated with increased GDM risk. Our results are supported by findings from others [30–32], although an increase in the GDM rate with higher fat intake was not consistently observed [33]. The composition of dietary fat may modify such relationships. For instance, some studies have suggested that animal or saturated fat consumption is a risk factor for GDM [30, 34], whereas we and others [31] found evidence of an association with total fat, but not saturated fat per se. Apart from a diet rich in fat, the only food group we observed to be significantly positively associated with an increase in GDM risk was dairy products. However, literature does not consistently confirm this relationship [7], and these findings are contradictory given that the incidence of type 2 diabetes mellitus in the general population seems to be inversely correlated with milk consumption [35]. Overall, evidence on the importance of specific dietary components and the potential protective effect of beneficial dietary patterns is limited and warrants further research before particular recommendations for primary care can be proposed [7].

Recently, the potential role of maternal mental health on the development of GDM has received growing attention [11]. In the present analysis, anxiety and depressive symptoms, or a generally low well-being in early pregnancy, all of which are early indicators for postpartum depression, did not significantly increase the women’s odds of developing GDM. Other studies previously suggested a correlation between depressive symptoms in pregnancy and GDM incidence, but data are thus far inconclusive [36, 37]. Importantly, we found in a previous analysis that both anxiety symptoms during pregnancy and GDM increased the risk for postpartum depression [38]. This is in line with observations from other studies [39].

Unlike previous investigations from large cohorts [9, 40], we could not demonstrate that other lifestyle factors, including smoking and education, had an impact on the risk of GDM. However, the investigators accounted for a less diverse set of pregnancy-related determinants in these studies [9, 40]. Smoking and educational level could represent surrogates of an overall unhealthy lifestyle, which may explain why our model, which comprised diverse lifestyle variables including diet and physical activity, showed no significant association with GDM risk.

Our comprehensive approach has some limitations. The power calculation was based on the primary outcome of the original intervention trial and secondary outcomes such as GDM or exploratory analyses on the risk for secondary outcomes were not taken into account. Due to the exploratory character, our analyses were not adjusted for multiple testing. In addition, the clinical relevance of some described associations between lifestyle factors and disease risk may be limited and thus needs to be interpreted with caution. Some of the included covariates were self-reported by study participants, including lifestyle data, mental health and pre-pregnancy weight. This limits their precision and could have resulted in an underestimation of their reported influence on glucose tolerance. Since our trial was embedded into the routine care system, GDM tests were performed in the participants’ gynaecological practices. Although the staff was instructed on how to conduct tests and measurements under standardised conditions [21, 22], variation between practices cannot be excluded. GDM incidence was nevertheless overall comparable to recently reported national data [2].

Despite these limitations, the public health design of the GeliS trial enabled us to obtain comprehensive data on diverse health and behavioural variables under real-life conditions in a primary care setting. Research focusing on GDM risk has mostly investigated limited risk factors rather than considering various lifestyle determinants. This is particularly important as the aetiology of GDM is complex and consists of the interaction between diverse variables. Including several potential determinants for GDM prediction is unique and a particular strength of our approach. We were able to assess the combined role of socio-demographic, health and lifestyle variables in one model. This is important in order to estimate the clinical relevance and to derive future strategies for primary care. In addition, the GeliS cohort will be followed-up for 5 years after pregnancy. This will enable us to investigate the long-term consequences of maternal glucose intolerance on early childhood outcomes and the interplay between diverse lifestyle factors.

In conclusion, maternal age and pre-pregnancy BMI seem to be the most important predictors of GDM. Normalising body weight prior to conception is probably the most powerful modulator of GDM risk, albeit difficult to achieve. Beyond that, lifestyle factors such as dietary fat consumption can play a role in GDM risk, but this warrants further research. Future research is needed to confirm which lifestyle factors are most salient in relation to GDM risk. This will provide the basis for supplying women with reliable recommendations aiming at reducing GDM risk at the individual level.

## Supplementary information

Supplementary Table S1 and S2

## References

[1] Ferrara A (2007). Increasing prevalence of gestational diabetes mellitus: a public health perspective. Diabetes Care.

[2] Melchior H, Kurch-Bek D, Mund M (2017). The prevalence of gestational diabetes. Dtsch Arztebl Int.

[3] Metzger BE, Lowe LP, Dyer AR, Trimble ER, Chaovarindr U, Coustan DR (2008). Hyperglycemia and adverse pregnancy outcomes. N Engl J Med.

[4] Nehring I, Chmitorz A, Reulen H, Kries R, von Ensenauer R (2013). Gestational diabetes predicts the risk of childhood overweight and abdominal circumference independent of maternal obesity. Diabet Med.

[5] Chiefari E, Arcidiacono B, Foti D, Brunetti A (2017). Gestational diabetes mellitus: an updated overview. J Endocrinol Invest.

[6] Brunner S, Stecher L, Ziebarth S, Nehring I, Rifas-Shiman SL, Sommer C (2015). Excessive gestational weight gain prior to glucose screening and the risk of gestational diabetes: a meta-analysis. Diabetologia.

[7] Schoenaker DAJM, Mishra GD, Callaway LK, Soedamah-Muthu SS (2016). The role of energy, nutrients, foods, and dietary patterns in the development of gestational diabetes mellitus: a systematic review of observational studies. Diabetes Care.

[8] Aune D, Sen A, Henriksen T, Saugstad OD, Tonstad S (2016). Physical activity and the risk of gestational diabetes mellitus: a systematic review and dose-response meta-analysis of epidemiological studies. Eur J Epidemiol.

[9] Solomon CG, Willett WC, Carey VJ, Rich-Edwards J, Hunter DJ, Colditz GA (1997). A prospective study of pregravid determinants of gestational diabetes mellitus. JAMA.

[10] Xiong X, Saunders LD, Wang FL, Demianczuk NN (2001). Gestational diabetes mellitus: prevalence, risk factors, maternal and infant outcomes. Int J Gynecol Obstet.

[11] Bowers K, Laughon SK, Kim S, Mumford SL, Brite J, Kiely M (2013). The association between a medical history of depression and gestational diabetes in a large multi-ethnic cohort in the United States. Paediatr Perinat Epidemiol.

[12] Kunath J, Günther J, Rauh K, Hoffmann J, Stecher L, Rosenfeld E (2019). Effects of a lifestyle intervention during pregnancy to prevent excessive gestational weight gain in routine care—the cluster-randomised GeliS trial. BMC Med.

[13] Günther J, Hoffmann J, Kunath J, Spies M, Meyer D, Stecher L (2019). Effects of a lifestyle intervention in routine care on prenatal dietary behavior-findings from the cluster-randomized GeliS trial. J Clin Med.

[14] Hoffmann J, Günther J, Geyer K, Stecher L, Rauh K, Kunath J (2019). Effects of a lifestyle intervention in routine care on prenatal physical activity—findings from the cluster-randomised GeliS trial. BMC Pregnancy Childbirth.

[15] Hoffmann J, Günther J, Stecher L, Spies M, Meyer D, Kunath J (2019). Effects of a lifestyle intervention in routine care on short- and long-term maternal weight retention and breastfeeding behavior-12 months follow-up of the cluster-randomized GeliS trial. J Clin Med.

[16] Hoffmann J, Günther J, Stecher L, Spies M, Geyer K, Raab R (2021). Infant growth during the first year of life following a pregnancy lifestyle intervention in routine care—findings from the cluster-randomised GeliS trial. Pediatr Obes.

[17] Rauh K, Kunath J, Rosenfeld E, Kick L, Ulm K, Hauner H (2014). Healthy living in pregnancy: a cluster-randomized controlled trial to prevent excessive gestational weight gain—rationale and design of the GeliS study. BMC Pregnancy Childbirth.

[18] Koletzko B, Bauer C-P, Bung P, Cremer M, Flothkötter M, Hellmers C (2016). Ernährung in der Schwangerschaft—Handlungsempfehlungen des Netzwerks “Gesund ins Leben – Netzwerk Junge Familie“. Frauenheilkd up2date.

[19] ACOG Committee Opinion No. 650. (2015). Physical activity and exercise during pregnancy and the postpartum period. Obstet Gynecol.

[20] Yaktine AL, Rasmussen KM. Weight gain during pregnancy: reexamining the guidelines. National Academies Press; Washington, DC, 2009. 20669500

[21] Kleinwechter H, Schäfer-Graf U, Bührer C, Hoesli I, Kainer F, Kautzky-Willer A (2016). Gestationsdiabetes mellitus (GDM)—Diagnostik, Therapie und Nachsorge. Diabetologie und Stoffwechs.

[22] Metzger BE, Gabbe SG, Persson B, Buchanan TA, Catalano PA, Damm P (2010). International association of diabetes and pregnancy study groups recommendations on the diagnosis and classification of hyperglycemia in pregnancy. Diabetes Care.

[23] Hedderson MM, Gunderson EP, Ferrara A (2010). Gestational weight gain and risk of gestational diabetes mellitus. Obstet Gynecol.

[24] Haftenberger M, Heuer T, Heidemann C, Kube F, Krems C, Mensink GBM (2010). Relative validation of a food frequency questionnaire for national health and nutrition monitoring. Nutr J.

[25] Kuhn D-A. Entwicklung eines Index zur Bewertung der Ernährungsqualität in der Studie zur Gesundheit Erwachsener in Deutschland (DEGS1), German (“Development of a dietary quality index in the German Health Examination Survey for Adults”). Robert Koch-Institut; Berlin, 2017.

[26] Chasan-Taber L, Schmidt MD, Roberts DE, Hosmer D, Markenson G, Freedson PS (2004). Development and validation of a Pregnancy Physical Activity Questionnaire. Med Sci Sports Exerc.

[27] Topp CW, Østergaard SD, Søndergaard S, Bech P (2015). The WHO-5 Well-Being Index: a systematic review of the literature. Psychother Psychosom.

[28] Kroenke K, Spitzer RL, Williams JBW, Löwe B (2009). An ultra-brief screening scale for anxiety and depression: the PHQ-4. Psychosomatics.

[29] Hui A, Back L, Ludwig S, Gardiner P, Sevenhuysen G, Dean H (2012). Lifestyle intervention on diet and exercise reduced excessive gestational weight gain in pregnant women under a randomised controlled trial. BJOG.

[30] Bowers K, Tobias DK, Yeung E, Hu FB, Zhang C (2012). A prospective study of prepregnancy dietary fat intake and risk of gestational diabetes. Am J Clin Nutr.

[31] Ley SH, Hanley AJ, Retnakaran R, Sermer M, Zinman B, O’Connor DL (2011). Effect of macronutrient intake during the second trimester on glucose metabolism later in pregnancy. Am J Clin Nutr.

[32] Saldana TM, Siega-Riz AM, Adair LS (2004). Effect of macronutrient intake on the development of glucose intolerance during pregnancy. Am J Clin Nutr.

[33] Radesky JS, Oken E, Rifas-Shiman SL, Kleinman KP, Rich-Edwards JW, Gillman MW (2008). Diet during early pregnancy and development of gestational diabetes. Paediatr Perinat Epidemiol.

[34] Park S, Kim M-Y, Baik SH, Woo J-T, Kwon YJ, Daily JW (2013). Gestational diabetes is associated with high energy and saturated fat intakes and with low plasma visfatin and adiponectin levels independent of prepregnancy BMI. Eur J Clin Nutr.

[35] Alvarez-Bueno C, Cavero-Redondo I, Martinez-Vizcaino V, Sotos-Prieto M, Ruiz JR, Gil A (2019). Effects of milk and dairy product consumption on type 2 diabetes: overview of systematic reviews and meta-analyses. Adv Nutr.

[36] Hinkle SN, Buck Louis GM, Rawal S, Zhu Y, Albert PS, Zhang C (2016). A longitudinal study of depression and gestational diabetes in pregnancy and the postpartum period. Diabetologia.

[37] Ross GP, Falhammar H, Chen R, Barraclough H, Kleivenes O, Gallen I (2016). Relationship between depression and diabetes in pregnancy: a systematic review. World J Diabetes.

[38] Johar H, Hoffmann J, Günther J, Atasoy S, Stecher L, Spies M (2020). Evaluation of antenatal risk factors for postpartum depression: a secondary cohort analysis of the cluster-randomised GeliS trial. BMC Med.

[39] Azami M, Badfar G, Soleymani A, Rahmati S (2019). The association between gestational diabetes and postpartum depression: a systematic review and meta-analysis. Diabetes Res Clin Pr.

[40] Bouthoorn SH, Silva LM, Murray SE, Steegers EAP, Jaddoe VWV, Moll H (2015). Low-educated women have an increased risk of gestational diabetes mellitus: the Generation R Study. Acta Diabetol.

