# Supplementary material for: How does antenatal lifestyle affect the risk for gestational diabetes mellitus? A secondary cohort analysis from the GeliS trial
Source: Eur J Clin Nutr. 2021 Apr 23;76(1):150–8. doi: 10.1038/s41430-021-00910-9 (PMC8766288; doi:10.1038/s41430-021-00910-9)
Supplement: Supplementary file 1 — Supplementary Table S1 and S2 [file 41430_2021_910_MOESM1_ESM.docx]

**Supplement**

**Supplementary Table S1** Associations between demographic and lifestyle factors and the odds of developing GDM – model including linear covariates

| **Covariate** | **Model 1** | **Model 2** | **Model 3** | **Model 4** |
| --- | --- | --- | --- | --- |
| Group allocation | **1.04 (0.76–1.43)** | **1.04 (0.75–1.42)** | **1.05 (0.77–1.45)** | **1.05 (0.77–1.45)** |
| BMI^*^ | **1.13 (1.10–1.17) ^+++^** | **1.13 (1.09–1.16)^+++^** | **1.13 (1.09–1.16) ^+++^** | **1.13 (1.09–1.16) ^+++^** |
| Age^*^ | **1.06 (1.03–1.10) ^++^** | **1.07 (1.03–1.11)^++^** | **1.07 (1.03–1.11) ^+++^** | **1.07 (1.03–1.11) ^+++^** |
| Parity^*^ |  | **0.96 (0.76–1.21)** | **0.92 (0.73–1.17)** | **0.92 (0.73–1.17)** |
| Early GWG^*^ |  | **0.99 (0.92–1.05)** | **0.98 (0.92–1.05)** | **0.98 (0.92–1.05)** |
| HEI score^†^ |  |  | **0.92 (0.76–1.11)** | **0.91 (0.76–1.11)** |
| PA^‡^ |  |  | **1.01 (0.99–1.04)** | **1.01 (0.99–1.04)** |
| Smoking^§^ (categorial) |  |  | **1.12 (0.81–1.55)** | **1.12 (0.81–1.55)** |
| Low education^\|\|^ (categorial) |  |  |  | **0.99 (0.63–1.56)** |
| Antenatal distress^¶^ (categorial) |  |  |  | **1.02 (0.74–1.41)** |

^*^effect sizes are calculated per unit of measurement respectively, ^†^effect sizes are calculated per 10 HEI points, ^‡^effect sizes are calculated per 10 MET-h/week, ^§^current or former smokers, ^||^general secondary education or lower, ^¶^PHQ-4 score of ≥ 3 points.

**^+^***p*<0.05, **^++^***p*<0.01, **^+++^***p*<0.001; BMI: body mass index, GDM: gestational diabetes mellitus, GWG: gestational weight gain, HEI: Healthy Eating Index, MET: metabolic equivalent of task, PA: physical activity, PHQ-4: Patient Health Questionnaire 4.

|  | **No GDM** | **GDM** | **Total** | **p value^*^** |
| --- | --- | --- | --- | --- |
| **Energy and macronutrient intake** | n=1504 | n=183 | n=1687 |  |
| Energy intake^b^ [kcal/day] | 1959.1 ± 633.0 | 1951.9 ± 674.4 | 1958.3 ± 637.4 | 0.507 |
| Fat [E%] | 26.5 ± 6.3 | 27.5 ± 6.6 | 26.6 ± 6.4 | 0.013 |
| Saturated fat [E%] | 12.5 ± 3.4 | 13.0 ± 3.5 | 12.6 ± 3.4 | 0.033 |
| Protein [E%] | 16.1 ± 3.2 | 16.4 ± 3.4 | 16.2 ± 3.2 | 0.289 |
| Sugar [g/day] | 54.7 ± 31.4 | 56.5 ± 39.5 | 54.9 ± 32.3 | 0.601 |
| Fibre [g/day] | 24.2 ± 10.5 | 23.0 ± 12.0 | 24.0 ± 10.7 | 0.012 |
| Low fat diet (< 30 E%) [n(%)] | 1063/1504 (70.7%) | 113/183 (61.7%) | 1176/1687 (69.7%) | 0.013 |
| Carbohydrates < 50 E% [n(%)] | 267/1504 (17.8%) | 46/183 (25.1%) | 313/1687 (18.6%) | 0.015 |
| **Food intake** | n=1656 | n=202 | n=1858 |  |
| Soft drinks [ml/day] | 235.8 ± 663.9 | 207.3 ± 569.5 | 232.7 ± 654.2 | 0.895 |
| Sweets and snacks [g/day] | 70.8 ± 58.9 | 72.1 ± 56.5 | 70.9 ± 58.6 | 0.977 |
| Dairy products [g/day] | 321.5 ± 328.1 | 376.5 ± 378.0 | 327.5 ± 334.2 | 0.012 |
| Meat and meat products [g/day] | 82.4 ± 58.7 | 81.2 ± 47.7 | 82.3 ± 57.6 | 0.611 |
| Fast food [g/day] | 47.5 ± 36.1 | 46.6 ± 36.0 | 47.4 ± 36.1 | 0.552 |
| **Physical activity**^‡^ | n=1644 | n=202 | n=1846 |  |
| Sedentary behaviour | 12.9 ± 11.2 | 14.3 ± 11.3 | 13.0 ± 11.2 | 0.028 |
| Moderate intensity physical activity | 49.8 ± 51.2 | 51.2 ± 53.3 | 49.9 ± 51.4 | 0.810 |
| Vigorous intensity physical activity | 1.5 ± 3.8 | 0.9 ± 2.6 | 1.4 ± 3.7 | 0.005 |
| Sports | 9.8 ± 9.6 | 8.5 ± 8.9 | 9.6 ± 9.5 | 0.039 |
| ^*^p value for differences between women with and without GDM, tested with χ^2^ test for categorial variables and Kruskal-Wallis test for continuous variables, ^†^mean ± SD (all such values), ^‡^[MET-h/week] | | | | |
| E%: energy percent, GDM: gestational diabetes mellitus, MET: metabolic equivalent of task. | | | | |

**Supplementary Table S2** Dietary and physical activity variables in study participants with positive and negative GDM diagnosis
